# Draft Genome Sequence of Streptomyces albidoflavus 09MW18-IS, Cultivated from the Atlantic Ocean off the Coast of Virginia

**DOI:** 10.1128/MRA.00872-21

**Published:** 2022-01-06

**Authors:** Indu Sharma, Masuud Washington, Jeremy Chen See, Rola Suleiman, Regina Lamendella

**Affiliations:** a Department of Biological Sciences, Hampton University, Hampton, Virginia, USA; b Thermo Fisher Scientific, Albany, New York, USA; c Juniata College, Huntington, Pennsylvania, USA; d University of Virginia, Charlottesville, Virginia, USA; University of Southern California

## Abstract

We report a draft genome sequence for Streptomyces albidoflavus strain 09MW18-IS, isolated from the Atlantic slope off the coast of Virginia. The whole-genome sequence will provide novel insights into biosynthetic gene clusters and ecological adaptation in an oligotrophic environment.

## ANNOUNCEMENT

*Streptomyces* species from marine environments are adapted to grow in the presence of seawater and can be found in sediments or in association with aquatic plants and invertebrates (1). The genus *Streptomyces* alone accounts for 80% of the natural products within the order *Actinomycetes* (2). Here, we sequenced the genome of Streptomyces albidoflavus 09MW18-IS, cultured from near-benthic seawater collected at a depth of 258 m (global positioning system [GPS] coordinates, 37°05′66.94″N, 73°51′13.06″W). The sampling site is located off the coast of Virginia along the Atlantic slope near Washington Canyon. The seawater sample was plated onto seawater complete medium as described previously (3). On day 10, a small pale-yellow colony with a powdery appearance was selected, and an axenic culture was obtained by streaking it onto solid artificial seawater medium.

Amplification of the 16S rRNA gene using the actinomycetes primers For243 (GGA TGA GCC CGC GGC CTA) and R1378 (CGG TGT GTA CAA GGC CCG GGA ACG) and sequencing confirmed that the isolate is a member of the genus *Streptomyces* (4–6).

Whole-genome sequencing was performed as described previously (3). Briefly, genomic DNA was extracted using the UltraClean microbial DNA isolation kit (Qiagen, Inc.) and sequenced using Illumina and Oxford Nanopore technology (ONT). For Illumina sequencing, the library was prepared using the Nextera XT library prep kit (Illumina, San Diego, CA), followed by sequencing using 150-bp paired-end chemistry on a NextSeq 550 platform. A total of 13,649,123 read pairs were generated. For ONT sequencing, the DNA was concentrated using a Vacufuge instrument (Thermo Fisher Scientific, Waltham, MA). The genomic DNA was neither sheared nor size selected. The library was prepared using the Nanopore rapid sequencing (SQK-RAD004) kit (Oxford Nanopore Technologies, Inc., Oxford, UK), with the protocol supplied by the manufacturer. Briefly, the DNA quality was checked using an Invitrogen Qubit 4 fluorometer and the 1× Qubit double-stranded DNA (dsDNA) high-sensitivity assay kit (Thermo Fisher Scientific). Adaptors were ligated and loaded onto the GridION R9.4.1 flow cell via the SpotON sample port. A total of 559,363 reads were generated with an *N*_50_ value of 7,797 bp. Poretools was used for base calling and demultiplexing (7); the adaptors were trimmed and the quality of the raw reads was checked using FastQC (8). However, error correction was not performed.

Trimmomatic v0.39 (9) was used for quality filtering and trimming the raw Illumina reads using the following parameters: LEADING:3 TRAILING:3 SLIDINGWINDOW:4:20 MINLEN:80. A total of 6,786,495 read pairs were retained, as well as a combined 4,881,460 individual forward and reverse reads. For all the following programs, default settings were used. Hybrid assembly was performed using SPAdes v3.13.0 (10) with the filtered short-read Illumina sequences and raw long-read Oxford Nanopore sequences, and the assembly summary statistics were generated using QUAST v5.0.2 (11). The total length of the draft genome is 7,118,966 bp; it is composed of 81 contigs (*N*_50_, 153,728 bp; *L*_50_, 13), with an average GC content of 73.18%. Recruiting the quality-filtered and trimmed reads back to the assembly using Bowtie2 v2.3.4 (11) yielded a mean genome coverage of ∼447-fold. The publicly available genome was annotated by Prokaryotic Genome Annotation Pipeline (PGAP) (12). The assembly contains 6,209 gene sequence predictions, 5,844 protein-coding sequences, 6 complete rRNA genes, 69 tRNAs, and 275 pseudogenes.

**FIG 1:**
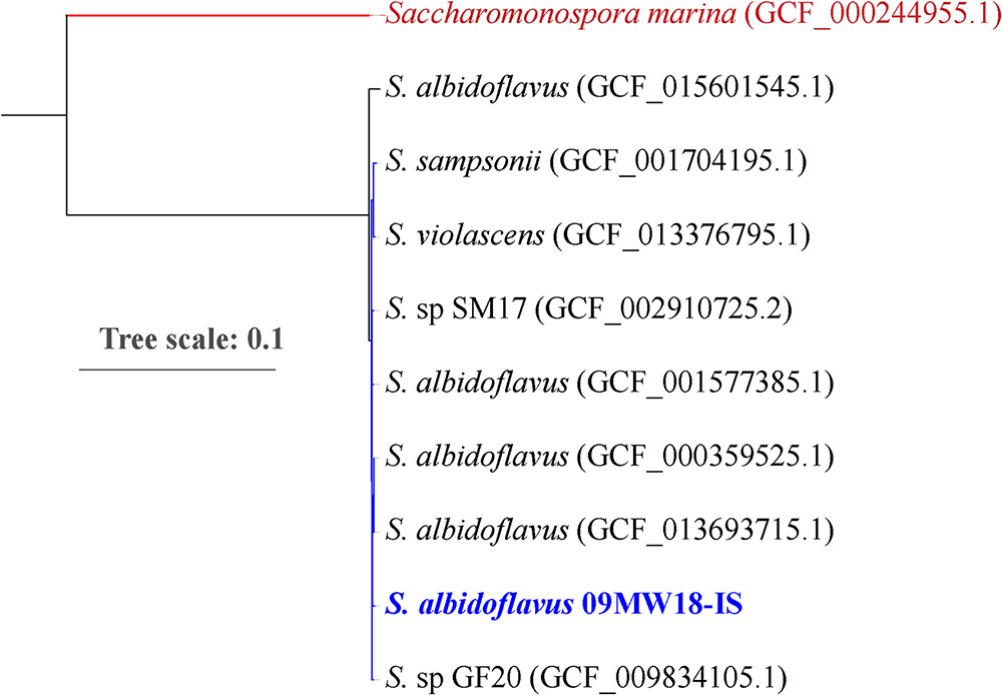
Estimated maximum-likelihood phylogenomic tree based on the concatenated amino acid alignment of 130 single-copy core genes specific for actinomycetes, with *Saccharomonospora* sp. as an outlier.

For taxonomic placement, GToTree v1.4.5 was used to create a phylogenomic tree of the nine closely related Streptomyces assemblies (Fig. 1) (13–18). Phylogenomics places *Streptomyces* sp. strain 09MW18 close to *S. albidoflavus* (GenBank accession number GCF_0013693715.1) and within the *S. albidoflavus* group (Fig. 1).

### Data availability.

This whole-genome shotgun genome assembly has been deposited at NCBI GenBank under accession number JAFRUC000000000.1. The raw sequencing reads have been deposited in the NCBI Sequence Read Archive under accession numbers SRX11958551 and SRX11958552 and BioProject accession number PRJNA562082.
